# Iris Antes 1969–2021

**DOI:** 10.1093/bioadv/vbac024

**Published:** 2022-05-04

**Authors:** Aphrodite Kapurniotu, Thomas Lengauer

**Affiliations:** 1 Division of Peptide Biochemistry, TUM School of Life Sciences, Technical University of Munich (TUM), Freising 85354, Germany; 2 Max Planck Institute for Informatics, Saarland Informatics Campus, Saarbrücken 66123, Germany

Dr Iris Antes, a professor at the Technical University of Munich, passed away on August 4, 2021 in Murnau near Munich. She was a productive scientist, a great mentor and an engaged activist for the causes of our field. Her untimely early death fills her friends, colleagues, mentees and students with great sadness.

**Figure:**
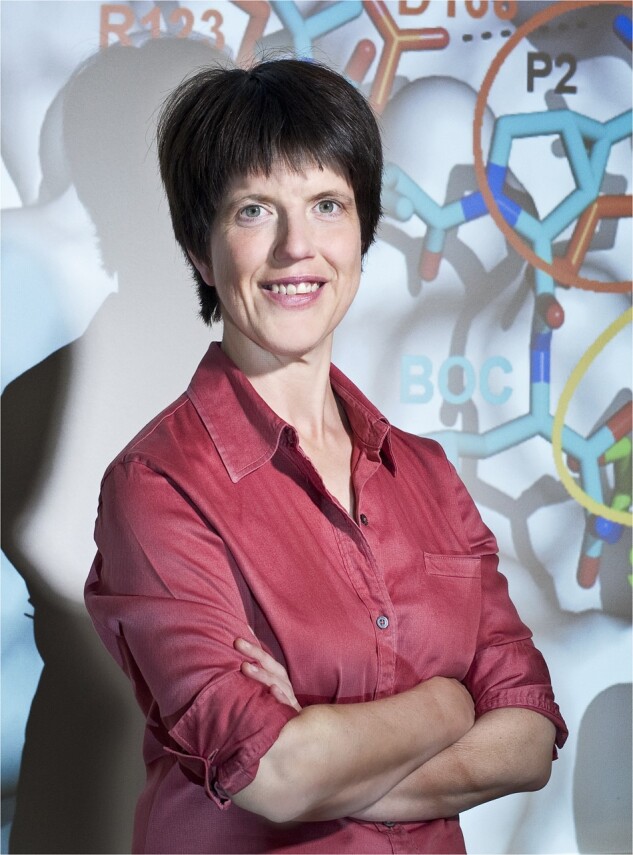
**Iris Antes**, credit: Astrid Eckert, Technical University of Munich

Iris Antes was born in Schwäbisch Gmünd on May 1, 1969. She studied chemistry at the Universities of Tübingen and Marburg and graduated with a PhD in Theoretical Chemistry at ETH Zürich in 1998 under the mentorship of Walter Thiel with a thesis on *Combined QM/MM Methods: From Link Atoms to Adjusted Connection Atoms*. Subsequently, she was a postdoc at the University of Pennsylvania with Michael Klein and at the University of California at Berkeley with David Chandler. From 2002 to 2008, she was a Junior Group Leader at the Max Planck Institute for Informatics (MPII). Within the Department for Computational Biology and Applied Algorithmics directed by Thomas Lengauer, she headed a group on computational protein modeling. Her work at the MPII addressed the structural analysis of molecular interactions involving proteins and, specifically, on modeling interactions between proteins and small ligands where the structural flexibility of the protein is taken into account. One of her main tools was molecular dynamics. Martin Zacharias explains: ‘One of Iris’ major research interests was to understand the mechanism by which biomolecules recognize and bind to each other. Such processes can involve significant conformational changes in partner molecules and her efforts focused on how to realistically and efficiently model such binding events in computer simulations. This led to the design of the DynaDock program (Antes, 2010) (and more recently its successor DynaBis; Melse *et al.*, 2022) that allow for efficient prediction of complex geometries formed by flexible partner molecules. The program has found various applications ranging from immune epitope docking (Antes *et al.*, 2006; Roomp *et al.*, 2010) to protein engineering and computer aided drug design (Ugur *et al.*, 2019)’.

Besides molecular dynamics, Iris also explored other approaches, for instance combinatorial optimization, which she pursued partially with her doctoral student Christoph Hartmann (Hartmann *et al.*, 2007). Part of her methodical work included parameter estimation in molecular modeling which she approached with inverse Boltzmann and machine learning methods (Antes *et al.*, 2005; Hartmann *et al.*, 2009). Besides basic methodical work and the development of useful software tools, she was also engaged in application studies, often with a pharmaceutical background (Voets *et al.*, 2005, 2006; Welsch *et al.*, 2008).

The memory of Christoph Welsch, a long-time collaboration partner, goes back to the first days of their meeting at the MPII: ‘We quickly recognized common interests and were both fascinated by the structure of proteins and their ability to adapt in multiple directions. Since then, we have worked together for many years until now, sometimes very closely. Our contact has never broken off, even after our time together at the MPII. Over the years, we performed exciting research on the biology of RNA viruses, in particular on drug resistance mechanisms in hepatitis C virus infection (Dultz *et al.*, 2020; Welsch *et al.*, 2015) and on mechanisms of viral fitness and immune escape (Welsch *et al.*, 2012). Our last paper is not finished yet and I was really looking forward to talk to Iris about the data—so I can’t believe that we will not be able to work together anymore!’.

In 2009, Iris completed her Habilitation at Saarland University with a thesis on *Computational Methods for the Investigation of Protein-Ligand Interactions*. In 2008, she moved to the School of Life Sciences at the Technical University of Munich on the newly created professorship for protein modeling. With joy and engagement, she dedicated herself to research and teaching in Theoretical Chemical Biology and protein modeling. She developed new computer-based methods for the elucidation of molecular processes, for drug design, protein engineering and computational immunology. Her research had translational character. Unfortunately, she will not be able to witness the results of such translation.

She engaged in many successful collaborations with colleagues in the scientific region of Munich and beyond and made valuable contributions to research projects of high repute. Her colleague Johannes Buchner remembers: ‘Within our collaborative research unit SFB 1035, a team of about 20 PIs, she set up and fostered joint projects with many different groups. A large number of publications resulted from these studies. One day, Iris was popping up spontaneously at my door excited about results of recent MD simulations and with some new ideas on how to approach our common project she wanted to discuss. Her scientific curiosity was never ceasing and she seemed to be immune against set-backs in the pursuit of her projects’. Martin Zacharias echoes this sentiment: ‘I co-organized two scientific retreats with Iris Antes. I remember that she was always very engaged in organizing and setting up a high-level and productive scientific agenda. In the case of technical difficulties or troubles concerning the organization she kept her optimism and never gave up searching for creative solutions good for everyone. She was also very positive and encouraging in her conversations and discussions with other scientists and her coworkers’.

Iris Antes was an outstanding scientist and academic teacher. Her colleagues appreciated her friendly outgoing and spontaneous character, and her high collaborative spirit. She was a dedicated mentor to her students. Her group members: ‘We felt her dedication while working with her and benefitted from her commitment. She encouraged us and our own ideas and helped us with great scientific advice. We are very grateful that she gave us the opportunity of working on our PhD and inspired us to advance in our development. We highly appreciate that she included us in an international group and community. We enjoyed various activities in the group like group seminars, winter schools and Christmas parties but also barbecues at the chair in summer, where we had the chance to get to know her on a personal level. And we are thankful that she made effort to create an enjoyable environment’. It is upon them now to carry further her ideas and her scientific legacy.

Iris was also engaged in internationally in supporting our scientific field. As one of the leaders of the Community of Special Interest (COSI) on Structural Bioinformatics and Biophysics (3DSIG) of the International Society for Computational Biology (ISCB), she directed a track at the flagship conference ISMB/ECCB 2021 of this society, even a few weeks before her death. She was active until practically the last minute. Rafael Najmanovich: ‘Iris was for many, many years one of the most active participants in 3DSIG, every year it was a joy to meet her and discuss the latest developments. I reached out to her to see if she’d be interested in co-organizing 3DSIG over a beer in Basel and she eagerly accepted. Since then Iris was instrumental in helping lead and organize 3DSIG. I remember in 2021, just two weeks prior to her untimely death, still discussing results while chairing a session at 3DSIG. Iris, with her subtle sense of humor and acute mind, will be deeply missed’.

Johannes Buchner: ‘She was very much looking forward to moving into our new building, the “Center for Protein Assemblies”. When I last saw her, she stopped by to check the status of her offices and told me she would be my neighbor in a few weeks. Unfortunately, that did not happen’.

We have got to know Iris as a dynamic, warmhearted, courageous, optimistic and exceptionally strong and independent person driven by great curiosity. In her leisure time, she loved to hike and climb on the mountains. Her passing leaves a gaping hole but her spirit and scientific work will be a driver for us into the future.

**Figure:**
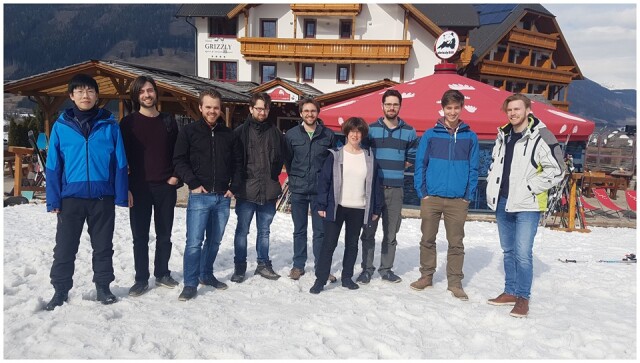
**Iris Antes with her team in the mountains in March 2018:** Left to right: Chen Zheng, Helmut Lutz, Okke Melse, Markus Schneider, Manuel Glaser, Iris Antes, Martin Zachmann, Lukas Wietbrock, Maximilian Meixner

## Funding

None declared.

*Conflict of Interest*: none declared.
